# Large-scale vivid metasurface color printing using advanced 12-in. immersion photolithography

**DOI:** 10.1038/s41598-022-18259-9

**Published:** 2022-08-18

**Authors:** Egor Khaidarov, Damien Eschimese, Keng Heng Lai, Aihong Huang, Yuan Hsing Fu, Qunying Lin, Ramon Paniagua-Dominguez, Arseniy I. Kuznetsov

**Affiliations:** 1 Institute of Materials Research and Engineering, A*STAR (Agency for Science, Technology and Research), 2 Fusionopolis Way, #08-03, Innovis, Singapore, 138634 Singapore; 2 Institute of Microelectronics, A*STAR (Agency for Science, Technology and Research), 2 Fusionopolis Way, #08-02, Innovis, Singapore, 138634 Singapore

**Keywords:** Optics and photonics, Optical materials and structures

## Abstract

Nanostructures exhibiting optical resonances (so-called nanoantennas) have strong potential for applications in color printing and filtering with sub-wavelength resolution. While small scale demonstrations of these systems are interesting as a proof-of-concept, their large scale and volume fabrication requires deeper analysis and further development for industrial adoption. Here, we evaluate the color quality produced by large size nanoantenna arrays fabricated on a 12-in. wafer using deep UV immersion photolithography and dry etching processes. The color reproduction and quality are analyzed in context of the CIE color diagram, showing that a vivid and vibrant color palette, almost fully covering the sRGB color space, can be obtained with this mass-manufacturing-ready fabrication process. The obtained results, thus, provide a solid foundation for the potential industrial adoption of this emerging technology and expose the limits and challenges of the process.

## Introduction

Color printing has gone a long way, from the early use of simple dyes and powders to the recent, and very fast-paced research in color generation at the nanoscale using optically resonant nanostructures^1^. In this emerging field, the material parameters (e.g. the permittivity and permeability and/or the absorption bands^2^), which define the appearance of macroscopic objects, concede to the nanoparticle characteristics (its size and geometrical shape) in determining its color, via the excitation of optical resonances^3^. Among the potential applications of nanostructured colors are high resolution color printing^4^, counterfeit control^5^, high end color displays^6^, filters, colorimetric sensing^7^ and information encoding^8^.

While nanostructure-based colors can be fade-free and have obvious advantages in terms of resolution, they also have the drawback of being sensitive to nanofabrication imperfections. In this regard, even a small variation in the size or shape of the particles might significantly change their scattering spectrum and, hence, the viewing color, thus requiring high-resolution nanopatterning techniques for their production. While state-of-the-art electron beam lithography (EBL) systems can indeed go well-beyond the needed precision^9^, fabricating the resonant nanostructures using high-throughput techniques is of out-most importance for practical applications^10,11^. This is one of the reasons why the initial attention paid to color generation using metallic particles exhibiting strong plasmonic resonances^12,13^ has gradually switched to the use of dielectrics, which not only have lower intrinsic losses^14^ resulting in narrower resonances and purer colors, but are also suitable for fabrication using well-developed industrial mass production processes. In this regard, Silicon (Si) is called to become the material of choice for its CMOS compatibility, together, but to a lesser extent, with other semiconductor materials, such as ${\text {TiO}}_{2}$^15–18^ or GaN^19^. For that, however, it is still necessary to prove that it can be used to produce the bright, vivid and highly saturated colors that have been obtained so-far by EBL (such as those providing full sRGB^20^ or even Adobe RGB^21^) or laser patterning^22^ but using mass-production-compatible techniques. In this direction, some works have taken the first steps towards establishing the use of deep UV photolithography for color reproduction in large sizes and volumes^23,24^. These initial results, however, have reported nanostructures and colors that are still one step behind in quality and coverage of those provided by EBL.

In this paper, we stretch the use of 12-in. immersion photolithograpy on silicon wafers and subsequent etching processes one step beyond to produce high-quality and high-throughput nanostructural colors that are closer to those done by EBL, thus bringing the technology closer to industrial applications. To do so, we optimized the processes and perform a detailed nanoscale characterization of the obtained nanoparticles, correlating them with the quantitative study of their color quality in the well-established sRGB color space.

## Results and discussion

**Figure 1:**
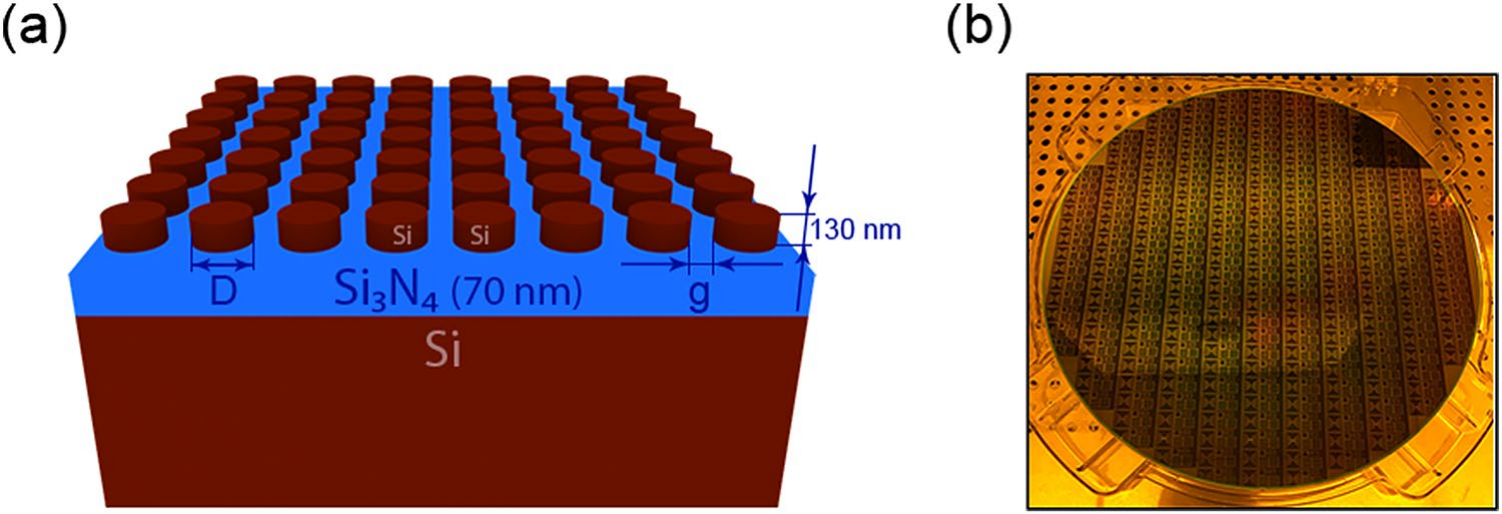
(**a**) Schematics of nanostructure design, (**b**) a photograph of the fabricated 12-inch wafer sample with photolithography mask.

**Figure 2:**
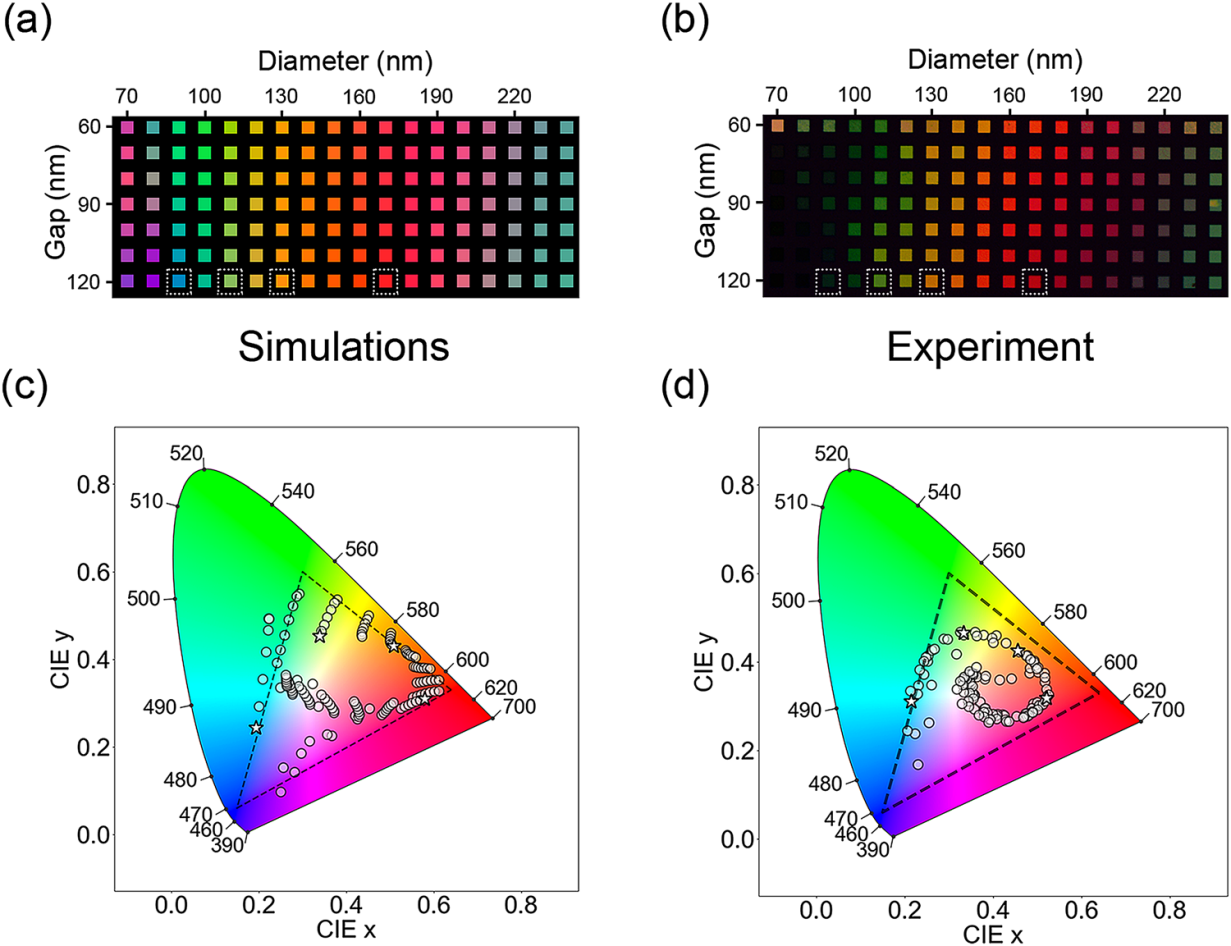
Numerical simulation (**a**) and experimental microscope imaging (**b**) results of the color palettes. CIE 1931 color diagram of numerical simulations (**c**) and experimental results (**d**). All the colors from the palette are marked as semitransparent circles; dashed triangle is the sRGB space. Dashed frames in (**a**) and (**b**) mark the colors for “N”, “S”, “L”, “M” letters corresponding to star markers in (**c**) and (**d**).

To have an insight into the color space coverage achievable, we designed and fabricated a discrete color palette of nanostructures. The overall nanostructure design schematics of palette elements are given in Fig. 1a (more details in “Methods”). A photograph of the fabricated by deep UV photolithography 12-in. wafer sample with photoresist mask of palettes is shown in Fig. 1b. In this palette, each element is formed by a square patch of a periodically repeated array, characterized by the nanodisk diameter (D) and gap (g) (Fig. 2a); the vertical axis corresponds to the nanodisk gap variation (g = 60–120 nm) and the horizontal one to the diameter variation (D = 70–240 nm), both with the step of 10 nm. These intervals have been chosen to provide a comprehensive coverage of the standard RGB (sRGB) color space without pushing the photolithography fabrication capabilities to the limit. For each of the palette elements we first simulated the reflection spectra. Each spectrum was then translated into the corresponding color and combined into the palette shown in Fig. 2a. Finally, all the spectra were convoluted with CIE color matching functions and displayed as white semitransparent circles in the CIE 1931 color space, as shown in Fig. 2c. Importantly, the designed colors cover the whole perimeter of the sRGB color space, which is shown as the dashed line triangle in the Fig. 2c.

The color palette was experimentally fabricated on a single sample and characterized optically, with more details given in “Methods” section. The recorded microscope image and the corresponding CIE plot for one of the samples are shown in Fig. 2b,d, respectively. Each color square in the palette corresponds to an arrays of 10 ${\upmu}{\text{m}}$ $\times$ 10 ${\upmu}{\text{m}}$ in size, and is equidistantly positioned 10 ${\upmu}{\text{m}}$ from the neighbouring squares. We note that, in experimental demonstrations of a single or few colors, the potential imprecision in the photolithography mask sizes can be compensated by simply taking a little larger or smaller dose. In contrast to that a good quality reproduction of the whole multicolor palette requires general calibration of all the arrays simultaneously. In our cases, this was achieved by including optical proximity correction (OPC) as a first step in the mask design.

The experimental results in Fig. 2 provide an immediate estimate on the quality of color reproduction using our process. It is important to note that the microscope image in Fig. 2b is the snapshot of the whole palette and illustrates the distribution of various reflection levels for various color patches, while Fig. 2a is a collection of patches each normalized to maximize brightness (as described in the procedures), hence they do not fully coincide; Fig. 2b has some dark areas where reflection is low. Fig. S1 with higher source brightness to reveal those colors is included in “Supplementary Information”. Optical images taken with higher numerical aperture objectives are included in Fig. S2. Detailed numerical simulations of oblique incidence impact (broadening and shift) on nanostructure spectra are shown in Fig. S3.

**Figure 3:**
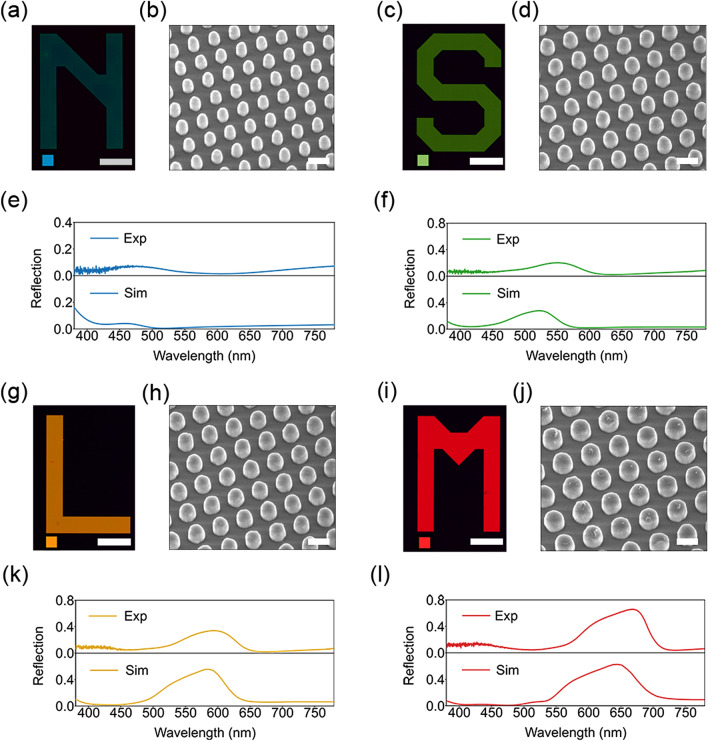
Microscope images of selected colors (**a**, **c**, **g**, **i**) with corresponding tilted-view SEM images of the nanostructures comprising each letter (**b**, **d**, **h**, **j**). The scale bars in the microscope images correspond to 200 $\mu$m, scale bars in the SEM images are 200 nm. The square color patches shown as insets in the bottom-left corners of the microscope images denote the target simulated colors. (**e**, **f**, **k**, **l**) show corresponding experimental (top) and simulated (bottom) reflection spectra for each of the arrays.

Importantly, the fabricated colors have an overall good coverage of the sRGB space. It should be noted, however, that some of the areas, specifically the corners of the CIE plot, are missing representative color points. Potential reasons for that include broader reflection peaks and additional side peaks in the spectra due to fabrication imperfections (see Fig. 3 for some examples). Another factor is the uniformity within a single color patch, which could be further improved if additional OPC for different patch areas is implemented. Finally, for further improvement, size correction could be implemented for the etching process as well. For some specific processes (such as the one used in this work) the etching can be nonlinear depending on nanostructure diameters, making smaller disk diameters be etched at a faster rate than the larger ones.

To demonstrate the quality and the scalability of the fabricated structures, we now focus on realizing several large-area features containing nanostructure designs providing characteristic colors spanning the sRGB range (the target colors are indicated in the palette plots in Fig. 2a,b by white dashed frames, corresponding to stars in CIE plots in Fig. 2c,d). Figure 3a,c,g,i show the optical microscope images of four different colored letters with sizes $> 0.5$ mm$\times$0.7 mm. The corresponding SEM images of the nanostructures comprising the letters are shown in Fig. 3b,d,h,j. The small square color patches displayed as insets in the bottom-left corners of the images represent the numerically simulated target colors. As can be seen there, we target vibrant tones of blue for the letter “N”, which is realized with nanodisks with gap g = 120 nm and diameter D = 90 nm (Fig. 3a,b), of green for “S”, using g = 120 nm and D = 110 nm (Fig. 3c,d) and of yellow (resp. red) for “L” (resp. “M”) using g = 120 nm and D = 130 nm (resp. D = 170 nm), as shown in Fig. 3g,h (resp. i, j). Panels (e, f, k, l) in Fig. 3 show corresponding experimental and numerical simulation spectra.

It can be readily observed that we were able to achieve a vivid green, yellow and red, with corresponding saturated and bright colors, and a dark background that indicates the good performance of the antireflective coating for these wavelengths even if only single layer is used. For yellow (“L”) and red (“M”) colors the experimental reflection maxima and shapes coincide well with the target simulations (Fig. 3k,l); target colors are well matched. The green “S” exhibits a slight redshift in reflection maximum resulting in different tone of green (Fig. 3f). The blue color (“N”) does not look as pure, becoming slightly darker and “greenish” as compared to the target one, that is easily explained by looking at the corresponding spectra (Fig. 3e). The experimental reflection maximum for “N” is wider, redshifted relative to simulations and has an additional reflection band in dark red. We attribute that to possible differences between simulation and experiment in the material properties, to slight deviations from circular cross-section in fabrication and to slight tapering introduced by the etch process, as observed from the SEM images. Additional details of tapering and fabrication defects are given in “Supplementary Information” (Figs. S4 and S5). For larger cylinders those experimental deviations have less impact on the spectra and, therefore, color quality, while smaller cylinders are significantly more sensitive. Furthermore, oblique incidence and large NA objectives as shown in Figs. S2 and S3 contribute to broadening and shift of the nanostructure spectra. Generally, resonant reflection bandwidth widening, shifts from colormatching functions maxima and additional side bands lead to less saturated, less vibrant colors. In corresponding color diagrams this translates to color points closer to the the middle white (Fig. 2c,d), having narrower gamut. While the experimental color gamut is narrower, essential sRGB space is generally covered. These results provide a glance into the potential of the developed process for practical applications of nanostructured colors.

## Conclusion

We demonstrated the potential of 12-in. immersion photolithography process for large scale industrial production of nanostructures for practical applications. The obtained colors are vivid, saturated, possess good variety of tones and provide general coverage of sRGB color space. Color palette is obtained in a single fabrication round, allowing for multistructural fabrication on a same wafer coupon. Furthermore, we pointed out several challenges of processes without pushing the limits of photolithography equipment resolution, generalizing the outcome for universal industrial adoption. Challenges include several OPC for single process fabrication of large scale multicolor palette combined with nonlinearity of the size reduction during etching process. With complementary spectral, angular incidence, tapering and SEM defect analysis we identified the possible sources of produced color deviation and ways of improvement. Results obtained here bridge perspective nanostructure color applications with industrial mass production capabilities. Low-cost, high-throughput photolithography process can become the drive for adoption of nanostructures in color printing, anticounterfeiting, color filters, high end color displays, instead of traditional dyes or thin films. Highly sensitive applications e.g. sensing or information encoding may require more development in terms of design for more spectrally robust nanostructures, while general fabrication framework and expected limits can be estimated from this work.

## Methods

### Design and numerical simulations

Figure 1a illustrates the schematics of the fabricated nanostructures on a 12-in. wafer. For the geometrical shape of the nanostructures, we chose periodic nanocylinders with circular cross-section for their polarization insensitive response and relative fabrication simplicity. These nanopillars are arranged in a square lattice and have a fixed height of 130 nm. Both inter-particle gaps and diameters are varied to excite, and spectrally shift, various resonances in the disks, hence generating different colors in reflection. The pillars are, additionally, positioned on top of an antireflective coating designed to minimize reflection from the Si substrate (and optimized for green wavelengths), which helps in expanding the generated color gamut. For that, a 70 nm silicon nitride ($Si_{3}N_{4}$) layer is considered on top of the *Si* wafer. The baseline for this structure design is adopted from Ref.^20^, and is re-optimized here via the corresponding geometrical tuning to account for discrepancies in the material dispersion. For that, the reflection spectra from homogeneous arrays of nanostructures are calculated using finite-difference time-domain method commercial software (Lumerical FDTD), with periodic boundary conditions (BFAST for oblique incidence) applied along the substrate surface and perfectly matched layers in the directions orthogonal to it. Plane wave excitation normal to the substrate is considered for palette calculations; oblique incidence case is described in “Supplementary Information”. The computed reflection spectra ($R(\lambda )$) are then converted to points in the standard CIE 1931 color space, as explained in further below. Tristimulus (XYZ) values are calculated by convolution of spectra $R(\lambda )$ with color matching functions ($r(\lambda ),g(\lambda ),b(\lambda )$).

1 $$\begin{aligned} X = \int _{380}^{780} R(\lambda ) r(\lambda ) d \lambda \qquad Y = \int _{380}^{780} R(\lambda ) g(\lambda ) d \lambda \qquad Z = \int _{380}^{780} R(\lambda ) b(\lambda ) d \lambda \end{aligned}$$

Then coordinates (x,y) for CIE can be found as:

2 $$\begin{aligned} x = \frac{X}{X+Y+Z} \qquad y = \frac{Y}{X+Y+Z} \end{aligned}$$

Target colors in sRGB space are obtained by XYZ2rgb function in scikit-image package for Python. Theoretical target colors brightness is maximized by normalizing the tristimulus values (XYZ) by their sum and then applying the XYZ2rgb function. In this case, the target colors have same maximum brightness and can be easily distinguished. This is done to clarify the target colors in case of low reflection levels for various color patches.

### Fabrication

The designed structures are experimentally fabricated on a 12-in. *Si* wafer using deep UV immersion photolithography and etching. The amorphous *Si* and $Si_{3}N_{4}$ layers are deposited on top of the Si wafer using the plasma-enhanced chemical vapor deposition (PECVD) method. A spin-on carbon (SoC) and an anti-reflection coating (ARC, for deep UV photolithography) layers are deposited on top of this stack to be used as a hard mask. The photoresist is deposited on top and processed using a 193 nm ArF immersion photolithography tool. Figure 1b shows a photograph of one of the fabricated 12-in. wafers containing the nanoscale color prints photolithography mask (before etching). The wafer with developed photomask is then diced into coupons and patterned using the following dry etching processes. Dry etch steps are shown in Fig. S4a. Photomask pattern is transferred to ARC layer using $CF_{4}$, $CH_{2}F_{2}$, $N_{2}$ etch and to SoC using $N_{2}$ and $O_{2}$ gas chemistry (LAM Exelan). Finally, Si is etched (SPTS Advanced Dielectric Etcher System) with $C_{4}F_{8}$, $SF_{6}$ and *Ar* gases at 20$^{\circ } C$. After that, the remaining SoC residue is removed using $O_{2}$ plasma. After fabrication, several selected nanostructures are imaged using a scanning electron microscope (SEM, Hitachi SU8220) to correlate the resulting sizes with the designed ones.

### Optical characterization

Finally, the nanostructures are optically characterized by measuring their reflection spectra using a microspectroscopy setup (inverted microscope Nikon Ti-U equiped with spectrometer Andor SR-303i). Dark current noise is subtracted from the obtained spectral signal followed by normalization to reflection of the aluminum film mirror. The sample is illuminated with a halogen lamp and the reflected light is collected using an objective with moderately low numerical aperture (NA = 0.5) as to mimic close-to-normal incidence. Microscope images of the color palette are also obtained using low magnification objective (Zeiss $5\times$, $10\times$ in Fig. 2b, $20\times$ with NA = 0.13, 0.2 and 0.4 correspondingly) and the brightness level is set to maximum possible, before the background color starts to deviate from black.

Both the numerically simulated and experimentally measured reflection spectra are convoluted with color matching functions and plotted on chromaticity diagrams.

## Supplementary Information

Supplementary Information.

## Data Availability

Data underlying the results presented in this paper are not publicly available at this time but may be obtained from the authors upon reasonable request.
