# Supplementary material for: Large-scale vivid metasurface color printing using advanced 12-in. immersion photolithography
Source: Sci Rep. 2022 Aug 18;12:14044. doi: 10.1038/s41598-022-18259-9 (PMC9388524; doi:10.1038/s41598-022-18259-9)
Supplement: Supplementary file 1 — Supplementary Information. [file 41598_2022_18259_MOESM1_ESM.pdf]

Supplementary information for:
Large-scale vivid metasurface color printing using advanced 12-inch immersion photolithography

Egor Khaidarov[1], Damien Eschimese[1], Keng Heng Lai[2], Aihong Huang[1], Yuan Hsing Fu[2], Qunying Lin[2], Ramon Paniagua-Dominguez[1], and Arseniy I. Kuznetsov[1,*]

[1]Institute of Materials Research and Engineering, Agency for Science, Technology and Research (A*STAR), 2 Fusionopolis Way,#08-03 Innovis, 138634 Singapore
[2]Institute of Microelectronics, Agency for Science, Technology and Research (A*STAR), 2 Fusionopolis Way, #08-02, Innovis, Singapore 138634, Singapore
[*]Arseniy_Kuznetsov@imre.a-star.edu.sg

High brightness color palette image

The optical microscope image of the color palette in the main text (Figure 2b) was taken with brightness level is set to maximum possible, before the background color starts to deviate from black. While it provides a general overview of color quality, some areas with low reflection remain dark and it is difficult to estimate the color. Figure S1 is a color palette image taken at higher light source brightness (same magnification x10 and NA = 0.2), revealing the colors in the left bottom corner. Although colors become brighter, Si_3N_4 layer reflection becomes observable: background deviates from target black and influences the color perception.

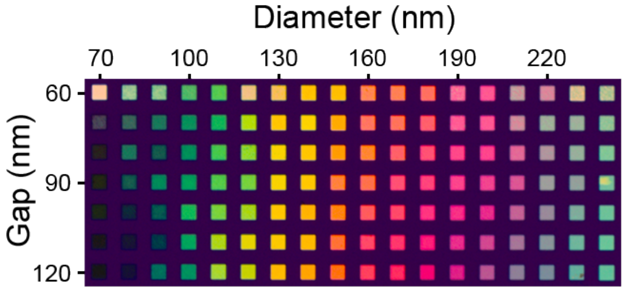

Figure S1. Optical microscope image of color palette taken at high light source brightness

Oblique incidence and large NA objectives

We have performed optical microscope imaging of colour palette to trace the color changes as a function of the objective numerical aperture (NA). Results are shown in Figure S2. NA = 0.13 (panel a) and NA = 0.2 (panel b) color palettes were taken as a one shot image, while NA = 0.4 (panel c) was stitched from two images to include all colors. Besides obvious difference in the resolution and sharpness of the image, colors do not significantly deviate, nonetheless, become slightly less vivid and saturated for higher NA.

Higher numerical aperture focuses/collects more angles of incidence, therefore we looked into behaviour of resonances at oblique incidence in order to explain the difference. Figure S3 shows numerical simulations of reflection spectra at various angles of incidence (β) for p-polarized light (electric field vector parallel to the plane of incidence). Angles of incidence β from

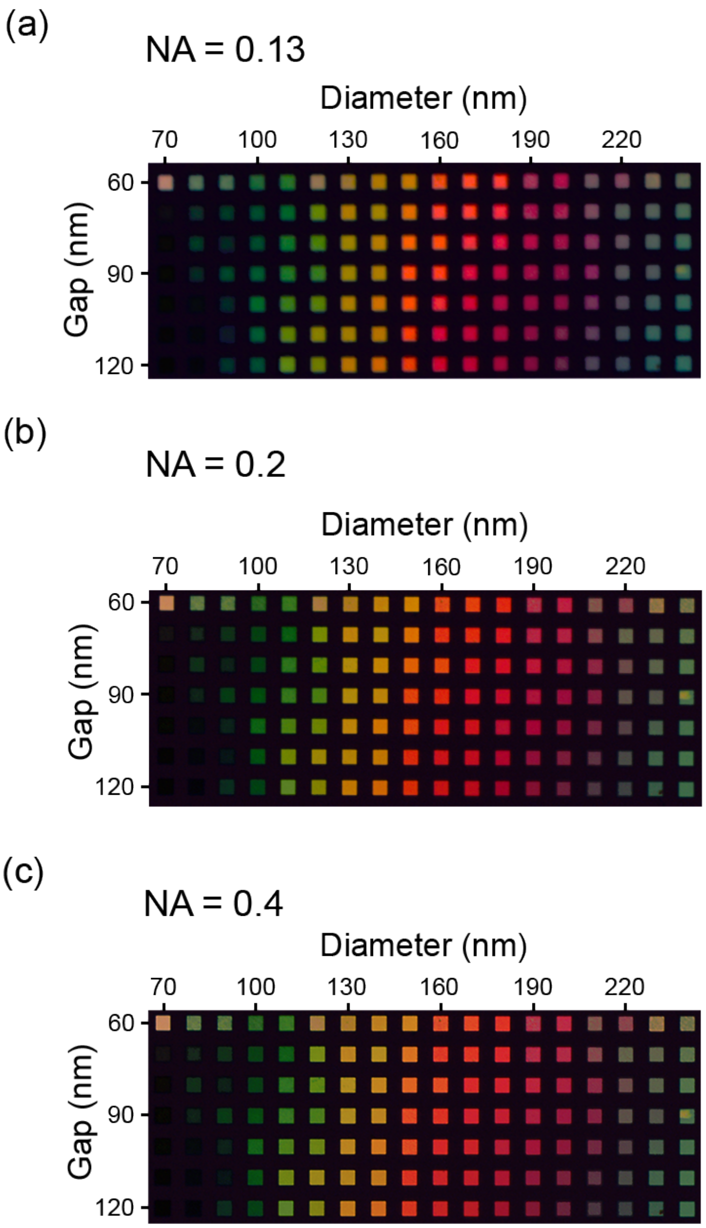

Figure S2. Optical microscope images of color palette taken with objective of different NA: (a) 0.13, magnification x5; (b) 0.2, magnification x10; (c) 0.4, magnification x20.

0° to 30° correspond to NA of the objectives used in this work (NA = 0.13 to 0.5). Panels (a) to (d) in Figure S3 correspond to reflection spectra of the selected designs in letters ”N”, ”S”, ”L”, ”M” (Figure 3 of main text).

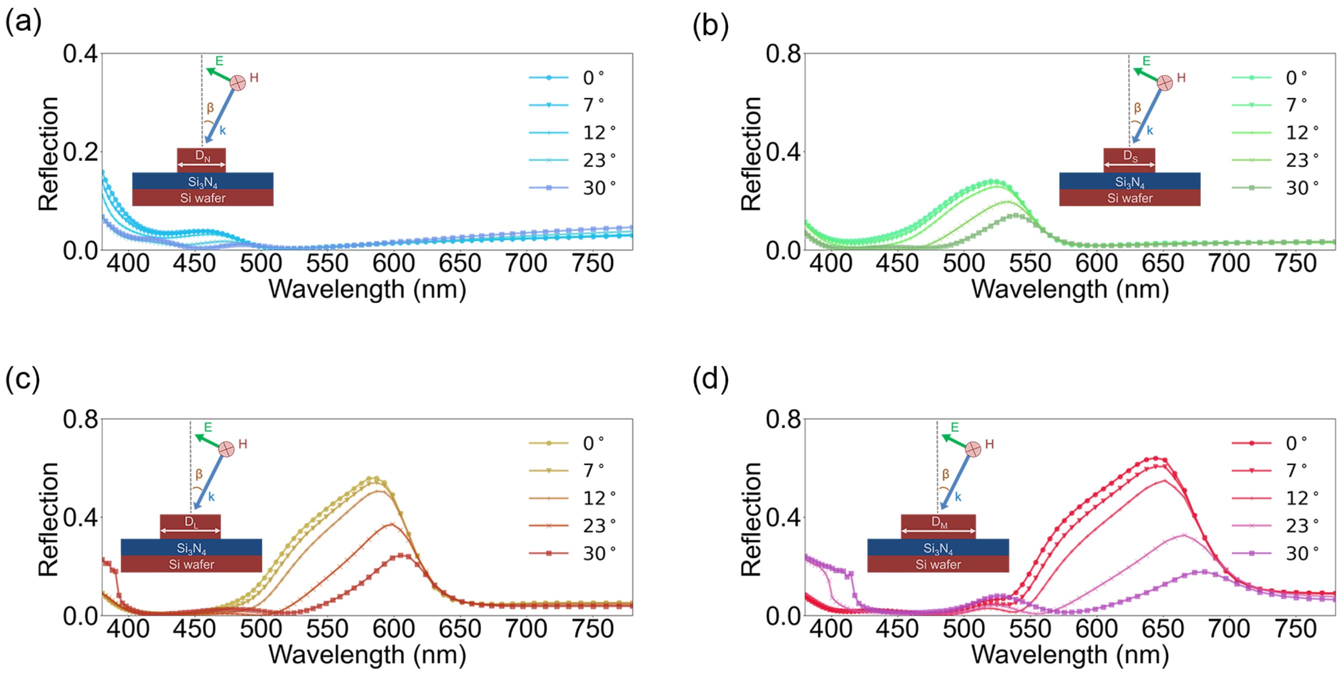

Figure S3. Numerical simulations of reflection spectra as a function of p-polarized light incident angle for ”N”, ”S”, ”L”, ”M” nanostructure designs (a,b,c,d correspondingly). Insets show structure schematics.

Figure S3 provides evidence of noticeable red shift relative to incidence angle for larger *Si* disks (”S”,”L”,”M” designs), with an exception of ”N” letter design where behaviour is more complex. Other polarization (s-polarization, electric field vector perpendicular to the plane of incidence) does not exhibit same behaviour as we increase the angle of incidence: spectra stays almost unchanged with negligible blue shift, therefore, we do not show it here. In the higher numerical aperture objective light is incident at all angles and the obtained reflection spectra is the result of the sum of those angles. Red shift of p-polarization as a result of oblique incidence can be one of the reasons of the experimental results red shift relative to numerical simulations spectra in Figure 3 in the main text. Numerical simulations there were estimated only for normal incidence. S-polarization oblique incidence may only contribute to slight broadening of the spectra.

Nanostructure shape analysis and tapering

Figure S4 demonstrates the fabrication flow (panel a) combined with the SEM images of nanostructure shapes evolution (panel b) after the following the fabrication steps: photolithography mask, etch of SoC mask and Si, etch mask removal (final structure), all fabrication details are given in Methods. SEM images are given from top and side/ 30° angle view relative to the sample surface. For evaluation we selected ”M” letter design (D = 170 nm and G = 120 nm). SEM images of photoresist mask expose small irregularities in the target circular mask shape, disk sizes were taken as an average of two orthogonal measurements. After SoC and *Si* etch a straight sidewall is observed, showing the quality of photoresist pattern transfer into *Si* nanostructures. Final image after SoC removal exposes a slight tapering from the *Si* etch estimated to be 4.6°, calculated from average bases and height of the truncated cone. Slight deviations from circular shape and tapering contributed into overall experimental spectra broadening relative to numerical simulations (Figure 3 of the main text).

Numerical simulations of nanostructure tapering effect on reflection spectrum are shown in Figure S5. Simulations were performed at normal incidence with tapering angle α up to 10°, almost double the estimated angle from experimental SEM images. In simulations the median diameter and height were fixed corresponding to design, varying angle determining the values of bases. Results demonstrate slight spectral broadening and blue shift of the tapered nanostructure resonances relative to cylinder shape. Therefore, we can conclude tapering and deviations from circular shape contributed into overall experimental spectra broadening relative to numerical simulations (Figure 3 of the main text).

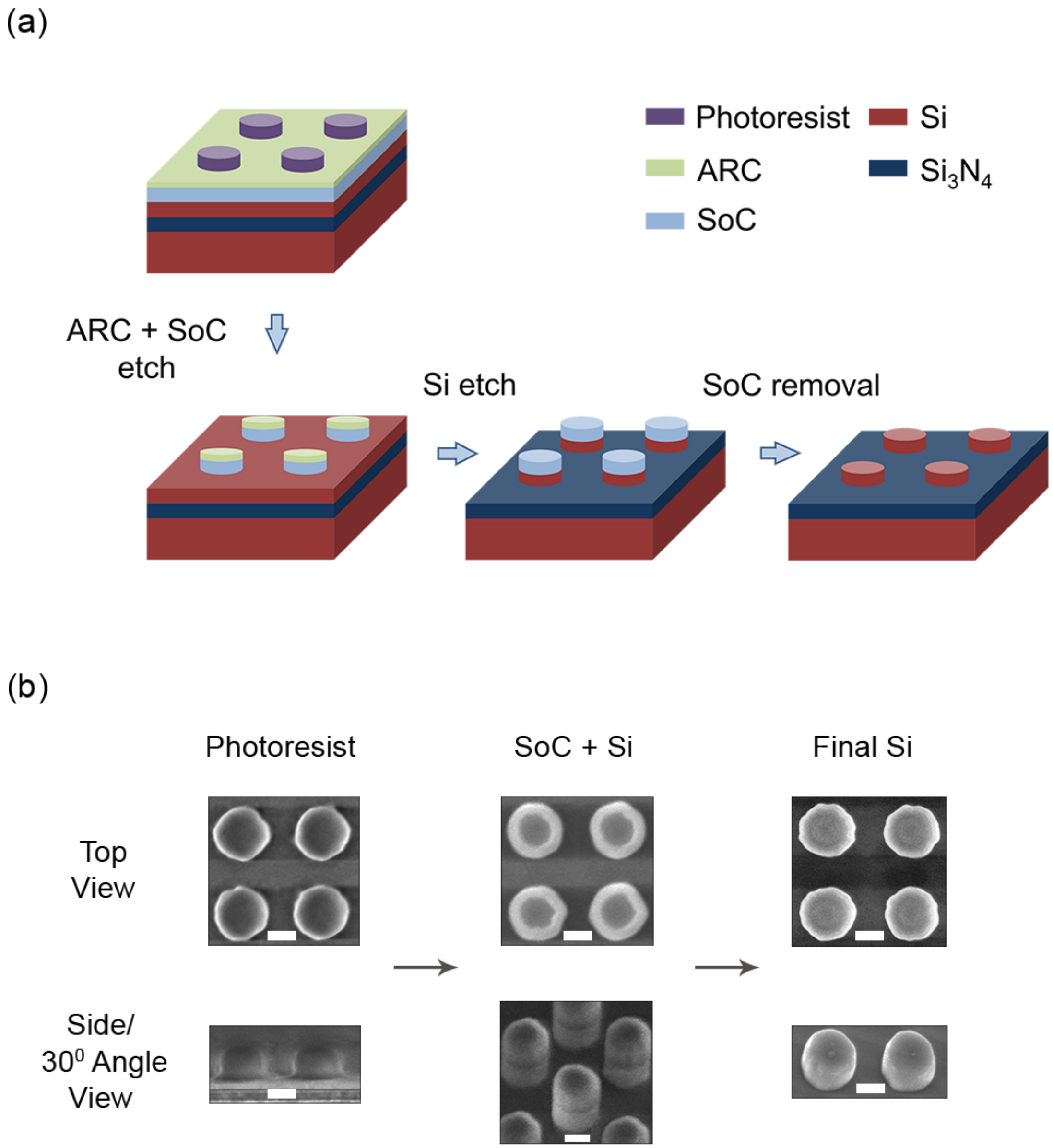

Figure S4. (a) Fabrication process flow schematics; (b) SEM images of nanostrusture shape after different fabrication steps: after photolithography development, after *SoC* and *Si* etch, after mask removal (final structure); scale is 100 nm.

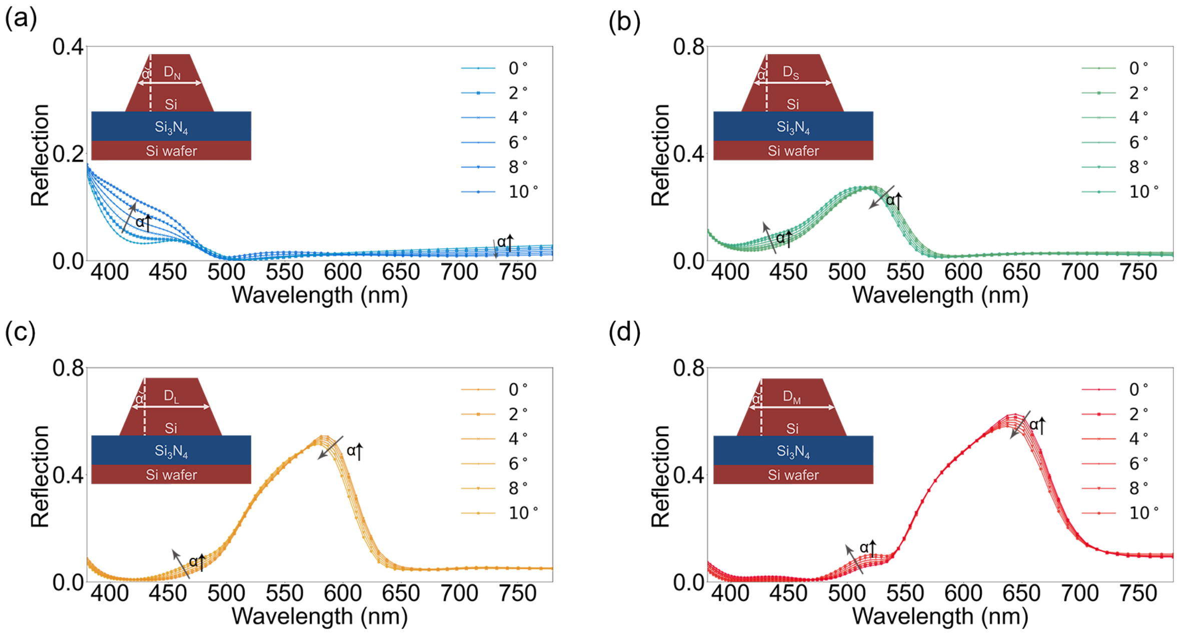

Figure S5. Numerical simulations of reflection spectra as a function of tapering angle (α) for ”N”, ”S”, ”L”, ”M” nanostructure designs (a,b,c,d correspondingly). Arrows show the plot shift direction as α increases. Insets show structure schematics.
